# Intelligent Tools to Monitor, Control and Predict Wastewater Reclamation and Reuse

**DOI:** 10.3390/s22083068

**Published:** 2022-04-16

**Authors:** Dimitris Ntalaperas, Christophoros Christophoridis, Iosif Angelidis, Dimitri Iossifidis, Myrto-Foteini Touloupi, Danai Vergeti, Elena Politi

**Affiliations:** 1 UBITECH Ltd., 15231 Athens, Greece; iangelidis@ubitech.eu (I.A.); vergetid@ubitech.eu (D.V.); epoliti@ubitech.eu (E.P.); 2 Greener than Green Technologies S.A., 14564 Athens, Greece; c.christophoridis@greenerthangreen.co (C.C.); d.iossifidis@greenerthangreen.co (D.I.); m.touloupi@greenerthangreen.co (M.-F.T.)

**Keywords:** wastewater reclamation, reuse, value-added compounds, decision support systems, artificial intelligence

## Abstract

Contemporary wastewater reclamation units entail several diverse treatment and extraction processes, with a multitude of monitored quality characteristics, controlled by a variety of key operational parameters directly affecting the efficiency of treatment. The conventional optimization of this highly complex system is time- and energy- consuming, frequently relying on intuitive decision making by operators, and does not predict or forecast efficiency changes and system maintenance. In this paper, we introduce intelligent solutions to enhance the operational control of the unit with minimal human intervention and to develop an AI-powered DSS that is installed atop the sensors of a water treatment module. The DSS uses an expert model, both to assess the quality of water and to offer suggestions based on current values and future trends. More specifically, the quality of the produced water was successfully visualized, assessed and rated, based on a set of input operational variables (pH, TOC for this case), while future values of monitored sensors were forecasted. Additionally, monitoring services of the DSS were able to identify unexpected events and to generate alerts in the case of observed violation of operational limits, as well as to implement changes (automatic responses) to operational parameters so as to reestablish normal operating conditions and to avoid such events in the future. Up to now, the DSS suggestion and forecasting services have proven to be adequately accurate. Though data are still being collected from early adopters, the solution is expected to provide a complete water treatment solution that can be adopted by a vast range of parties.

## 1. Introduction

Recently, major global drivers such as water shortage, climate change, lack of high-quality freshwater sources and cost issues related to water abstraction [1], have led to the gradual increase in water reuse [2]. In 2011, reclaimed water after treatment comprised 0.59% of total global water use [1], which is projected to reach 1.66% by 2030, surpassing desalination [1], with California, Singapore and Japan leading the way for innovative technological solutions in the area of water reuse [3]. Apart from the dire need to increase water reuse for environmental reasons, there is a significant financial incentive to exploit the certain valuable wastewater components, using state-of-the-art reclamation techniques, while serving the sustainability goals set by the circular economy pillars.

The food processing sector is not only extremely water-consuming [4,5] but also follows wasteful practices, discarding vast amounts of water and nutrients in the form of wastewater, which is difficult and costly to manage. However, it is an excellent source of valuable nutrients and organic value-added compounds (VACs) [6] with a variety of possible uses and benefits.

Conventional wastewater treatment techniques long-established in the food processing sector mainly include filtration, coagulation [7] and (primary/secondary) biological treatment [8]. They require high investments, skilled personnel and increased maintenance costs, providing slow processes, an extremely low capacity to adapt to wastewater changes and limited efficiency to degrade a high range of organic substances [9]. Such techniques offer an outdated approach to wastewater management, overlooking the potential to recover VACs and recycle/reuse treated water.

On the other hand, modern water reclamation units could integrate a multitude of highly adaptive and modular treatment techniques, including hybrid complementary wastewater treatment techniques such as membrane filtration, biological degradation and advanced treatment (photocatalytical/photolytical degradation) enabling the reclamation of VACs and the subsequent treatment and reuse of polluted wastewater. The units could include numerous sensors and data-monitoring instrumentation, controlled by multiple operational parameters affecting the quality characteristics of treated water, which could have a wide range of applications (water for irrigation, coolant, production water), frequently restricted by environmental and health regulations.

This introduces a high level of system complexity, especially in the cases where diverse wastewater treatment processes are employed, complementing each other. Up to this point, the optimization of the treatment processes has been mainly carried out through the manual evaluation of key parameter changes and the monitoring of selected indicators in a single-factor fashion. This approach is time- and energy-consuming, difficult to rapidly adjust to parameter changes and frequently relies on intuitive decision-making by operators. Additionally, it does not predict or forecast efficiency changes and system maintenance, in order to plan for operation/downtime. This workflow is quite inefficient in highly advanced treatment processes, which include several parallel or serial processes, with multiple scenarios of possible treated water applications. Advanced data mining and decision support tools are necessary to address optimization of procedures in complex wastewater treatment plants (WWTPs).

To address the complexity of the system, several prediction modeling techniques have been employed in the past [10,11,12,13,14]. Artificial Intelligence (AI) technologies, such as machine learning, deep learning and data analytics, have opened the way for intelligent decision making in wastewater management. Beyond the use of traditional methods, AI systems can further enhance the precision of optimal solution prediction, endowing existing applications with several degrees of intelligence. Numerous AI models have been successfully implemented for the removal of various pollutants from water [15]. For example, artificial neural networks (ANNs), fuzzy logic (FL) and genetic algorithms (GA) are typical AI approaches for solving multivariate nonlinear problems and thus have been modeled using experimental data to simulate, predict, confirm and optimize contaminant removal in wastewater treatment processes [16]. Although AI technologies are powerful tools for wastewater treatment, there are several limitations that hinder their greater application. For instance, such methods require sufficient data for experimental training, testing and overfitting of the whole process, which can itself be quite challenging. Moreover, sudden fluctuations in the input variables or changes in the operating parameters may tamper with the results and thus impair the precision of the predictions.

The design, planning and evaluation of water treatment processes requires the implementation of a decision support tool (DST) to evaluate alternative treatment techniques (conventional or advanced) and WWT trains, as well as water reuse applications [2]. Up to now, factors affecting the design and planning of a WWT process have included costs, energy consumption and effects of wastewater quality on human health (legislation and effluent guidelines). A holistic decision support system (DSS) allows integrating all the issues and provides a framework to solve multi-scenario problems [17]. DSSs have estimated, in the past, the cost of WWT by using life cycle cost analysis (LCCA) for the economic evaluation of alternatives. This is a complex task, taking into account financial, environmental and social criteria. Therefore, DSSs have employed multi-criteria decision-making approaches (most often the analytical hierarchical process (AHP) [18] to aggregate different criteria, including strengths and limitations for each WWT process and technique [2,19,20]. In the past, more elaborate multi-criteria analyses (MCAs) have been used to make comparative assessments of diverse WWT approaches, taking into account several criteria in real decision making, regarding the selection of the most sustainable wastewater treatment (WWT) technology [21], or intelligent-knowledge-based systems and superstructure-based optimizations. In this case, a mathematical model is employed and integrated in the DSS to generate a solution for water treatment under various water reuse scenarios [22].

Nevertheless, the need to simultaneously optimize design and energy consumption, enhance operation and improve effluent quality and environmental sustainability requires the use of advanced intelligent DSS (IDSS), using data-driven approaches, such as environmental data mining [11,17] in the area of environmental data science [23,24]. For this purpose, the analysis of online sensor data of increased quality [25], the integration of measures to address micro-pollutants [26] and to valorize VACs present in the wastewater [27] and the participation of end users in the operation and design of fit-for-purposes WWT systems [24] are essential and integral parts of an IDSS and necessary to develop a water reclamation process highly adaptive to rapid changes.

Although data prediction algorithms and DSS systems have been developed in the past, their integration to produce a holistic AI-powered system requiring minimal human intervention has not been thoroughly studied, especially in the sector of food processing. Additionally, the introduction of criteria, regarding the optimum reclamation of VACs, in combination with consequent wastewater treatment, has not been attempted. The information derived from online process monitoring through sensors, treated-water quality prediction modeling and operation simulation of the water reclamation process can be integrated in an IDSS fed by internal and external criteria to produce possible water reuse applications.

In this study, we propose a novel treatment approach that is based on combining a water treatment unit with an AI-powered DSS. The solution aims to introduce intelligent solutions to enhance the operational control of the unit, with minimal human intervention and to develop an iDSS, utilizing knowledge acquisition to control the selection of optimal treatment conditions and to forecast data, aiming to minimize downtime. The unit will provide decision support to end users (farmers, food processing industry, VAC consumers, etc.), thereby reducing or eliminating the cost associated with estimating a strategy for treating water waste. In this manner, businesses that follow a traditional water treatment disposing technique may adopt state-of-the-art recycling and reclamation techniques without having to pay the additional cost of an expert. This has both a positive financial effect as well as an environmental one.

The outcome is expected to aid the food processing sector in becoming less water-intensive, to help it transition from a linear (take, make and waste) approach towards a more circular business model and to ultimately improve its overall environmental footprint. Moreover, the exploitation of VACs can provide an additional source of income, while the increase in the production of VACs will improve their availability on the market, and their health benefits will become more accessible to the wider public. The transition towards a circular and sustainable business model enables the formation of useful synergies at a regional and local level.

The overall structure of the paper is as follows: Section 1 consists of the introduction, while Section 2 outlines the main theory behind the WTM operations, as well as the main outputs of the DSS together with a description of the techniques employed to achieve them. Section 3 lists the results and the evaluation of these results from an initial set of early adopters consisting of one food processing company and three individual farmers. Section 4 summarizes the main conclusions of the present work and lists the next steps for the further development and evaluation of our solution. Finally, Abbreviations provide a table of the acronyms that are used throughout the present work and their descriptions.

## 2. Materials and Methods

**Figure 1:**
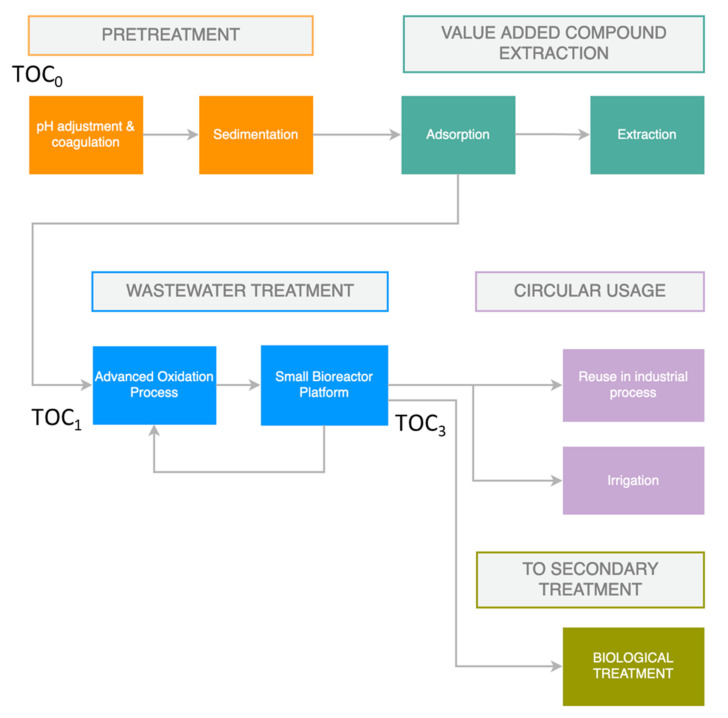
Diagram of the water reclamation process.

In the water reclamation process (Figure 1), a multitude of highly adaptive and modular treatment techniques are employed, which enable the reclamation of VACs and the subsequent treatment and reuse of polluted wastewater. The recovery of VACs is initially carried out using a combination of sorption/extraction techniques, followed by purification. Subsequently, complementary wastewater treatment techniques are employed, including advanced treatment (photocatalytical/photolytical degradation) followed by biological degradation. Each process is a separate modular unit able to be applied separately or complementary to each other depending on the wastewater qualitative and quantitative characteristics, the targeted treated water uses (e.g., irrigation, reuse in the production cycle, further treatment, etc.) and the available budget.

In the proposed methodology, there is the need to minimize human intervention and provide mechanisms for quick and targeted decision making and response. To address this challenge, innovative deep learning approaches, advanced data analysis methods and predictive analytics will be applied to the usage, health and performance data derived from the units to empower the current approach with intelligent analytical and decision support tools for predictive maintenance (early detection of malfunctions and minimization of the downtime), optimization of the WWT process (fine tuning of the various parameters for improvement of the final output), sustainability (energy saving, reusability of the various outputs) and commercial opportunities leading to financial benefits (identification of various outputs of significant market value, unknown before, energy savings, etc.).

**Figure 2:**
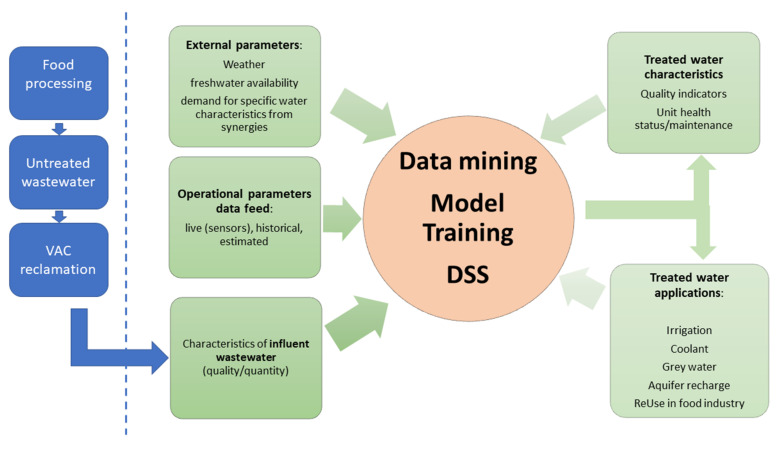
Flow chart of the proposed methodology.

The workflow of the process is depicted in Figure 2. The DSS is fed by various types of information: static knowledge originating from domain expertise, past experience, external data such as weather data and running irrigation water prices, historical data and real-time information. The latter originates from sensors measuring key parameters of wastewater, such as turbidity, pH, total organic carbon (TOC), specific UV absorbance at 254 nm (SUVA254), total suspended solids (TSS) and temperature. The accuracy of the previously mentioned sensors is ±1%, 0.1%, 2%, 3%, 5% and 0.05%, respectively. These are the major parameters monitored during the system operation. At the same time, the operation of the system is controlled by several factors, mainly including the amount of coagulant, contact time in the treatment unit (flow rate), number of oxidizing agents in advanced oxidation unit (AOP), flow rate of HCl or NaOH necessary to change pH, intensity of irradiation source and dissolved oxygen in the mix.

The treated water can be potentially used in many different ways (application scenarios in Figure 2), including for irrigation for nearby fields, as a coolant, as reused process water, as aquifer recharge, etc., depending on external parameters such as water demand, water prices, water scarcity due to weather conditions, etc.

Using deep learning, the DSS is able to provide suggestions and real-time recommendations both in the backward (goal-driven) and in the forward (data-driven) sense. More specifically, in the forward sense, the DSS can monitor the measured sensor values and, using forecasting algorithms, predict the future values of the variables being monitored. This information can be used by the DSS models to assign a rate to each of the possible uses of the wastewater (e.g., for irrigation, coolant, etc.) together with an estimation of how these ratings will evolve in the immediate future. Backward reasoning, on the other hand, allows for the specification of the desired usage (e.g., irrigation); the DSS will then give a rating of how good the monitored wastewater is for the desired usage along with suggestions of possible treatment actions to improve the quality of wastewater to meet the goal demands.

Although the prototypical treatment unit and the DSS have been implemented, data are still being collected by early adopters. The present work documents the methodology followed in Section 2 and gives the first qualitative results that were gathered by the early adopters in Section 3.

Details of the DSS are given in the following subsection.

### 2.1. AI-Powered DSS for Water Treatment

For facilitating semi-automatic decision making, a DSS prototype has been implemented. The original prototype was developed in the context of the DigiCirc accelerator program [28]; the work being presented here expands upon the prototype to include a number of extended models that involve external forecasts, such as weather forecasts and financial data.

**Figure 3:**
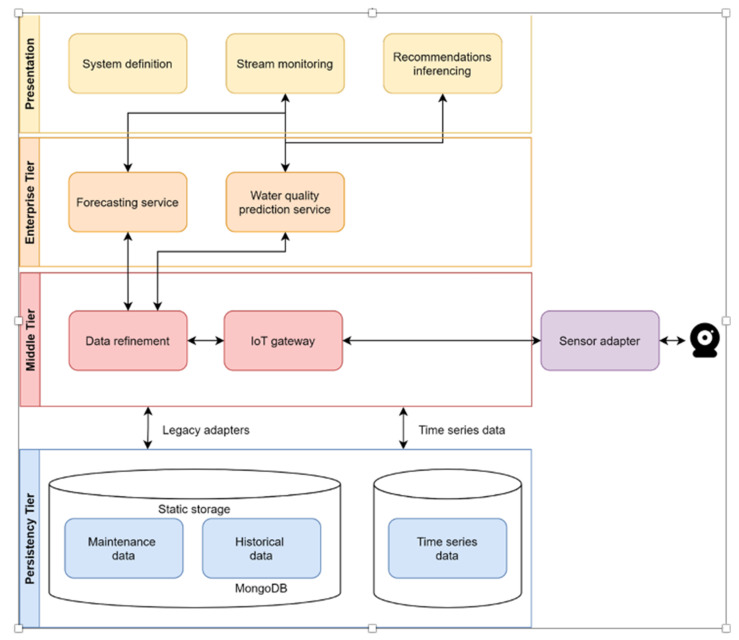
DSS Overall architecture.

The overall architecture of the DSS can be seen in Figure 3. Summarily, the main tiers and their corresponding functionalities are:

- Persistency Tier: the tier where all data are stored.
- Middle Tier: the intermediate tier, which is responsible for transforming/providing data to the enterprise tier and storing data in the Persistency Tier. It is also responsible for obtaining data from various third-party sensors.
- Enterprise Tier: the tier containing the provided DSS services of the solution.
- Presentation Tier: the tier responsible for presenting efficient views of the data obtained by the enterprise tier.

This is an architecture typical of many applications that leverage legacy and IoT data to provide end users with prognostics and analytics. The powerhouse of the DSS lies in the two modules of the enterprise layer, namely:

- The water quality prediction service. This is used to represent, assess and rate the quality of the water based on a set of input operational variables.
- Forecasting service. This service is used to forecast future values collected via sensors.

Working in tandem, the above modules provide full DSS functionality to the end user; based on the quality prediction service, the user can gauge the best course of action based on the latest status, while forecasting the estimation of the evolution of said quality can be also used for enhancing decision support. The end user may opt for the best action by taking into account all relevant parameters; for example, she/he may opt to ignore an optimum course of action if the forecasting services suggest that the overall cost may be minimal if the decision is deferred.

The methodology for implementing the above two services is listed in the following sections.

### 2.2. Water Quality Prediction Service

Briefly, water quality can be represented by a series of factors directly measured or estimated by the expert user; these include turbidity, TOC, pH and SUVA254. Assessing the quality of water and deciding the best treatment method typically involves expert opinion. The quality prediction service aims to eliminate the need for this human intervention by modeling this decision process. The model works both in the forward and in the backward sense.

In the forward sense, the aim is to generate a cost estimation for each possible action and to provide this to the end user. To that end, we employ a logistic regression model [29], which takes as input the aforementioned parameters as a single state and, combined with the selling value of the water (which, for now, is provided by manual uploads by the user), and with the current weather forecasts, as these are provided by open weather services (OpenWeather), predicts the quality of the processed water. The quality is classified into one of three categories: very good, very bad or acceptable, according to the well-known formula of calculating the logit transformation of the probability of the presence of the characteristic of interest, which is given by:

$$logit\left(p\right)=b_{0}+b_{1}x_{1}+b_{2}x_{2}+\cdots +b_{k}x_{k}$$

with the weights are calculated by fitting the model to the training data.

In the backward sense, the aim is to estimate the operational variables based on a target outcome that the end user inputs. The typical use case scenario for this functionality is when the end user wishes to exploit VACs in a specific manner (due to, for example, current market prices) and gauges the water treatment module to check in which range the observed values must lie to make the target goal profitable. For the backward sense reasoning, the DSS employs the Skope Rules algorithm. Skope Rules [30] combines the interpretability of a decision tree [31] and the modelization power of a random forest [32]; it partitions the variable space in a way that incorporates both expert opinion and fitting based on training data.

**Figure 4:**
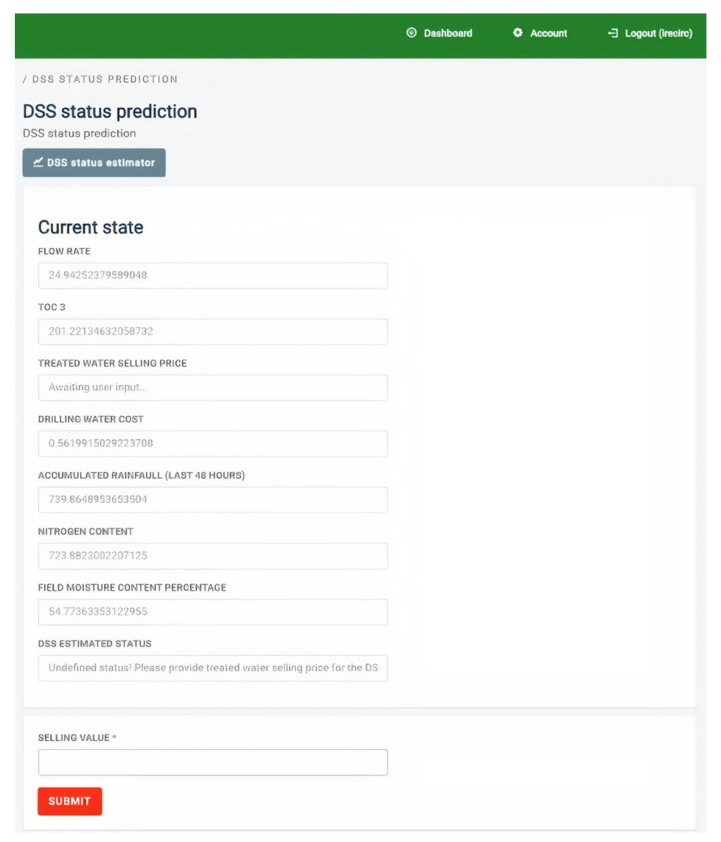
Water quality prediction module. Values are given by end user or collected by the IoT device connected to the water treatment module.

For the prototype of the water quality prediction module, as this is exposed to the end user under a prototypical implementation of the DSS’ GUI, see Figure 4.

### 2.3. Sensor Forecasting Service

The purpose of the forecasting of sensors is two-fold:

- To ensure that risks can be averted in time. For example, if, during processing, a variable reaches a critical level, it could negatively affect the entire pipeline.
- To provide the water quality prediction module for computing costs and optimum actions for the future, thus facilitating decision making, taking into account both current values and future trends. In this sense, the DSS can be used to find minima of cost values that take into account the future evolution of the system; these minima may be more efficient than local minima computed by taking into account only the current values, as these are recorded by sensors.

To implement the module, we employ two models of the same family, which differ slightly in their purpose: an autoregression model [33] and a vector autoregression (VAR) model [34].

Both models function in a similar fashion, so the main idea behind their operation will be illustrated here for both. The models treat the provided input as time series data. Given the past values of a specific sensor(s) and a sufficiently large rolling window through time, the models predict the values the sensor(s) will produce in the future. It is important to note that all time steps between samples must be the same, meaning if a value was not received by a sensor for a specific time step for some reason, it should be replaced by a default value. Both models were trained with lags = 1.

Sensor forecasting was implemented for the multivariable case. The single-variable case was used to make predictions of future values of sensors based on data recorded exclusively on said sensor. For many variables, such an approach may be sufficient, especially if the values recorded correspond to variables that have low correlations with other aspects of the treatment module. The multivariable case was used to make forecasts of variables that had strong correlations. This facilitates both the more accurate predictions of variables that may have multiple dependencies and forecasting of variables that are difficult to measure directly, by extracting their trend based on calculations performed in trends of other variables, which are easier to monitor.

For the prototypical implementation of the DSS and for the single-sensor case, the output to the user comes in the form of a graph. The graph consists of two main parts, the red part and the blue part. The red part illustrates the observed sensor values of the past, which also serves as training data for the estimator, while the blue part is the forecasted values. The graph is very dynamic in nature, as it allows (among other things) the following interactions:

- Inspection of the exact sensor value at any given time step.
- Thumbnail of the entire time series.
- Arbitrary zoom in/out of the time series by controlling the rolling window of the thumbnail, which in turn affects the area displayed in the main graph.
- Fine-grained range control for the monitoring window. Default monitoring windows of 5, 10, 20, 30 and 60 min and even the complete graph are provided as options.
- Ability to download the graph as a PDF, CSV and PNG.

For the multivariable case, though fitting and training of the models are more complex, the output is very similar, with the main difference being that all correlated variables can be monitored simultaneously.

**Figure 5:**
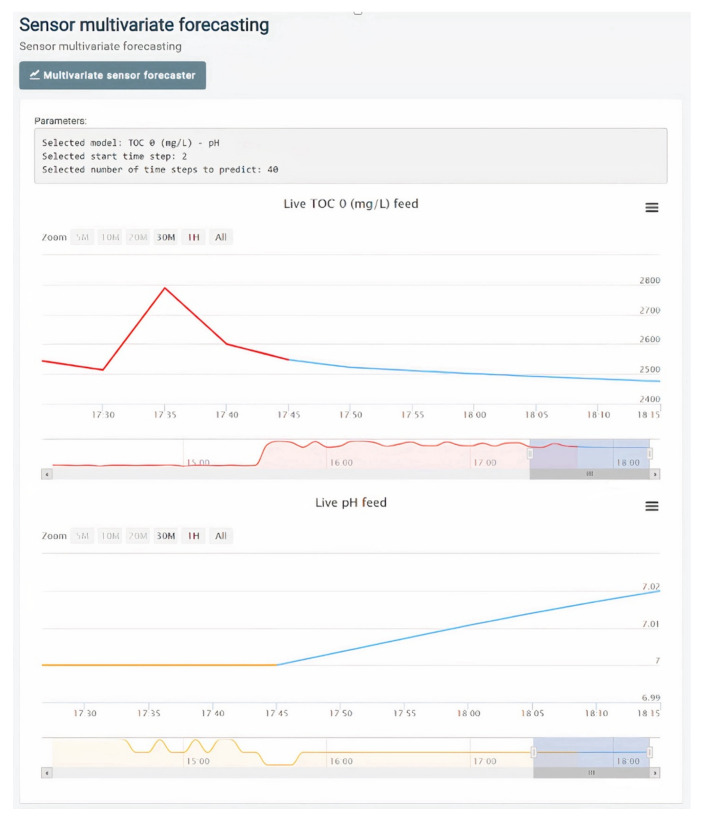
Multivariable forecasting, depicted for the TOC measured at the input of the module and the pH of the water. Red (yellow) lines correspond to the values measured for TOC(pH), with blue lines depicting the forecasts of the values.

Figure 5 depicts the monitoring of the sensor data for the multivariable case, with an example case of monitoring TOC values in conjunction with pH values; the graph for single-sensor forecasts is a special case of this graph with only one variable being depicted.

## 3. Results

Preliminary results of our methodology were collected by the usage of the water treatment module (WTM) and the accompanying DSS from a set of early adopters. Early adopters consisted of a food processing company and three individual farmers. The aspects evaluated were:

1. The extent to which important events could be extrapolated by the WTM and the underlying DSS alerting mechanisms.
2. Automatic responses of the WTM when measuring variables that are outside the thresholds constituting normal operations.
3. Validity of the DSS suggestions after evaluation of the end users.

These aspects will be described in the following subsections, with the aggregated results of the evaluation provided in a separate subsection.

### 3.1. Event Identification

By using the forecasting services of the DSS, important alerts can be generated when real-time measurements indicate that measured variables fall outside margins that correspond to normal operations. For the cases of single variables, this has been achieved by monitoring services of the DSS, which can easily generate alerts in the case of observed violation of operational limits. A more interesting case, however, is the event of dependent variables, where the multivariate forecasting can gauge disruptions and lead the WTM to adopt corrective actions as needed. An example of this is the total organic carbon (TOC) of the water before AOP treatment (Figure 1), which we designate as TOC_1_. When this is high, an oxidizing agent such as H_2_O_2_ in the AOP unit must be injected to lower the value. Using single-variable forecasting only, the DSS may take some time to adjust the trend when a disruption (e.g., input water with high TOC) occurs. If we perform multivariate forecasting, however, using the TOC measurements from water entering the WTM (which we indicate as TOC_0_), the disruption may be detected earlier, thus leading to more timely interventions.

**Figure 6:**
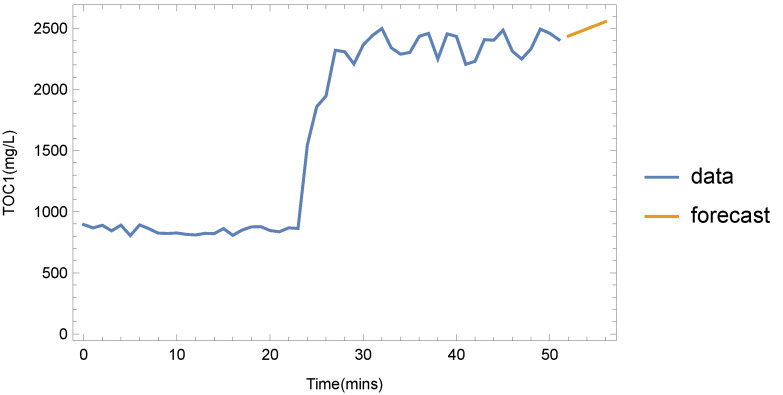
Forecasting the T0C_1_ values using single-variable method (data points are separated by 1 min intervals).

**Figure 7:**
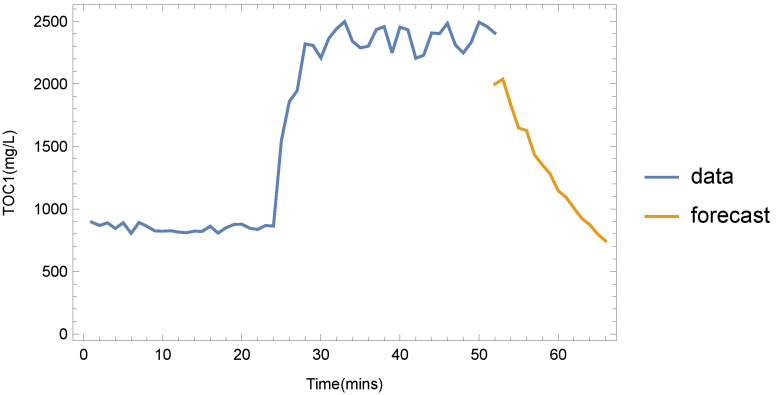
Forecasting the T0C_1_ values using a single-variable method (data points are separated by 1 min intervals).

These two cases are depicted in Figure 6 and Figure 7. In this example, there was a sudden increase in TOC_0_ after approx. 22 min of normal operation. After the 50 min mark, TOC_0_ was returned to normal levels. For the case of single-variable forecasting, the DSS projected high future values, as recent TOC_0_ values were not taken into account. The multivariate forecast, on the other hand (Figure 7), shows that TOC_1_ values are expected to fall. Although the exact values may still be misaligned from the real ones for some time, the event that denotes that TOC_1_ values have dropped can be easily deduced.

### 3.2. Automatic Responses

**Figure 8:**
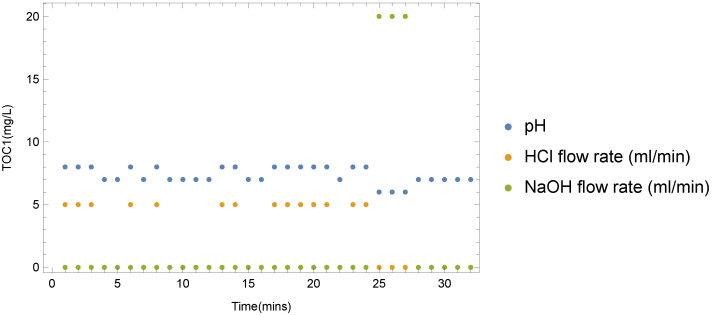
HCl and NaOH sensor values depicted against measured pH over a 30 min interval.

By constant monitoring of operational values, the WTM can react to changes without the need for human intervention. One such example is the automatic injection of HCl or NaOH to compensate for deviation in pH from the neutral value of 7. Figure 8 depicts a typical use case of the automatic responses feature. Each data point represents the observed values of pH, HCl or the NaOH flow rate in a minute interval. It can be seen that the responses of the WTM have a minimal (lower than one minute) latency in injecting HCl or NaOH to adjust the pH value.

### 3.3. DSS Suggestions

The DSS can offer suggestions via the generation of recommended actions by taking into account both current operational values, as these are recorded from sensors, and external input such as accumulated rainfall and the selling price of treated water. The rainfall currently originates from data offered by the OpenWeather service, while selling prices are inserted manually. Table 1 depicts the set of rules, and the recommended actions for each set of conditions. TOC3 denotes the total organic carbon content of the treated water.

DSS suggestions are evaluated in two aspects:

- Current aspect: in this aspect, the rules table is consulted and the recommendation is generated based on current values as these are inputs or measured directly from sensors.
- Prognostic aspect: In the prognostic aspect, forecast techniques are used to predict future evolution of time series data and the DSS offers the best recommendation, taking into account all future values to offer the best recommendation for the desired frame of reference. For evaluation purposes, a two-hour time frame was used; that is, the DSS recommended the best action based on estimation about the water contents and weather data over a two-hour period.

### 3.4. Overall Evaluation

The WTM and the accompanying DSS were evaluated using the aspects defined above and by collecting input from one company and three farmer units over the course of two weeks. These early adopters provided input for each one of the major aspects presented above, with an evaluation being performed every two hours. Evaluation took place by rating:

- Event Identification: Whether the DSS correctly predicted imminent events (such as TOC1 disruptions, sudden pH increases, etc.). A value of “Yes” denotes correct prediction of upcoming event, “No” denotes that an event observed was not predicted by the DSS, and the label of “Irrelevant” corresponds to either false positives or predictions that came too late to be of practical importance. Analysis also showed that the multivariable approach led to an increase in accurate event identification by an overall factor of about 21.2% when compared to single-variable forecasting. This rough figure was not measured directly, since end users only used the prototypical algorithm of the multivariable forecasts, but it was projected retroactively, based on sample reconstructions of a small subset of events and the relevant forecasts using single-variable forecasts.
- Reaction Validation: whether the WTM reacted timely to observed or forecast pH values.
- DSS Suggestion: the extent to which the DSS produced recommendations deemed correct by the users, based on the current observed values
- DSS Forecast: the extent to which the DSS produced future recommendations that were deemed correct by the users.

**Figure 9:**
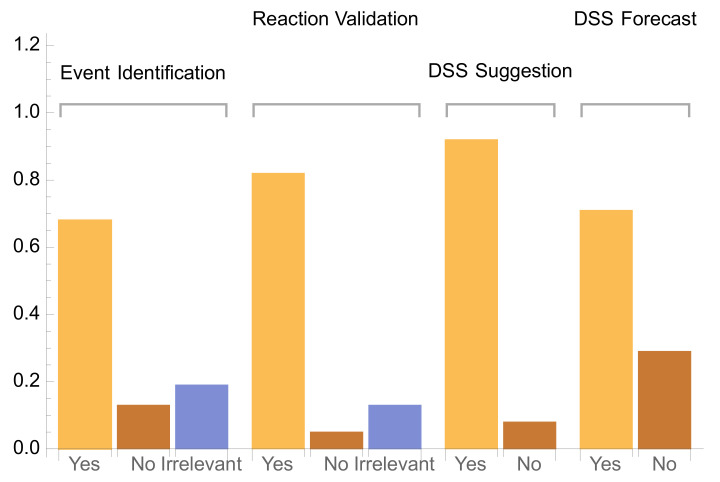
WTM and DSS preliminary evaluation results of early adopters.

The results of evaluation are depicted in Figure 9. Analysis of the results, together with post-experiment discussions with the involved adopters, led to the following preliminary conclusions:

- For the case of event identification, the DSS performed adequately, with most of the important events being correctly identified within the required timeframe. The “Irrelevant” responses correspond mainly to false positives, while around 13% of the events were not identified by the DSS. Post-experiment analysis of the data produced showed that some other combinations of variables for the multivariate forecast could produce better accuracy; this is an ongoing investigation whose results will undergo a second phase of early adopter evaluation.
- For the reaction validation, the majority of the cases were correctly anticipated by the DSS.
- For the DSS suggestion, the majority of recommendations were correct, with ~11% being wrong recommendations. This was anticipated, as the rules of the DSS are extracted from domain experience and the current state of the system is reliably represented by the sensor values and external parameters.
- For the DSS forecast, which was expected to improve upon the current predictions based on the DSS suggestion aspect, the overall accuracy of ~71% was significantly lower. Although this is a negative result, we have observed that from the wrong responses of the DSS suggestion module, a total of ~70% were correctly classified by the DSS forecast module.

## 4. Conclusions

In this paper, we presented an integrated solution of a water treatment module together with a DSS that could facilitate the adoption of complex water treatment procedures by small companies or farming units, overcoming the difficulties associated with the need for expert personnel. To this end, automation and expert opinion were embedded into the sensors of the WTM and the DSS recommendation engine, which allowed the generation of custom recommendations and the constant monitoring of the treated water.

The quality of the produced water was successfully monitored, assessed, rated and forecasted, using selected input parameters in single- or multiple-variable modes. The DSS was able to identify unexpected events whenever variables exceeded the range of operational limits. Finally, the DSS succeeded in implementing changes (automatic responses) in operational parameters in order to reestablish normal operating conditions. Based on different desired scenarios of water use and external parameters such as water prices, weather conditions, etc., the DSS provided suggestions and forecasting services with adequate accuracy.

Preliminary results from early adopters show that the output generated by the WTM and DSS is correct in the majority of the cases. Future work consists in the enhancement of the forecasting aspects of the DSS, especially the dynamic DSS engine and the multivariate forecasts needed for event identification and future recommendation, and the validation of this second version by a bigger set of early adopters.

## Figures and Tables

**Table 1:** DSS Rules.

Flow Rate (m^3^/h) AND/OR TOC_3_ (mg/L C)	Selling Price of Treated Water/m^3^	Usage Cost/m^3^ Drilling Water	Accum. Rainfall Last 48 h (mm)	Nitrogen Content mg/L	Field Moisture Content (%)	Recommendation
>1 AND <6.6	<0.7	>0.7	<1	0–1000	<15	Irrigation of nearby fields
>0.2 AND (1–20)	<0.7	>0.7	0–1000	>10	0–100	Irrigation of greenhouses
<100AND (5–10)	<0.5	0.5–0.7	>10–1000	<10	0–100	Reuse by company (cooling)
(any) AND (10–30)	<0.5	0.5–0.7	>10–1000	<10	0–100	Reuse by company (washing)
(any) and (6.6–500)	<0.5	0.5–0.7	>10–1000	1–10	0–100	Regional biological treatment

## Data Availability

Data will be available upon request.

## Abbreviations

List of Acronyms

**Acronym**	**Description**
AI	artificial intelligence
ANN	artificial neural network
AOU	advanced oxidation unit
DSS	decision support system
IDSS	intelligent decision support system
pH	potential of hydrogen
SUVA254	specific UV absorbance at 254 nm
TOC	total organic carbon
TSS	total suspended solids
VAC	value-added compounds
WTM	water treatment module
WWT	wastewater treatment
WWTP	wastewater treatment plant
